# Effects of Different Packaging Methods on the Quality of Fresh Red Apricots During Simulated Transportation and Storage After Transportation

**DOI:** 10.3390/foods15122068

**Published:** 2026-06-08

**Authors:** Jiale Zhang, Chengjie Wang, Meiyue Zhang, Yunfeng Pu, Yanju Xiang

**Affiliations:** 1 College of Food Science and Engineering, Tarim University, Alar 843300, China; 16637423287@163.com (J.Z.); wcj15731083671@163.com (C.W.); 19999764316@163.com (M.Z.); yfpu@zju.edu.cn (Y.P.); 2 Production & Construction Group Key Laboratory of Special Agricultural Products Further Processing in Southern Xinjiang, Alar 843300, China

**Keywords:** red apricot, packaging methods, simulated transportation, postharvest quality, physiological metabolism, cell wall ultrastructure

## Abstract

Three packaging methods were applied to fresh red apricots: P1 (plastic basket), P2 (breathable foam box), and P3 (perforated corrugated carton). To evaluate the effects of different packaging methods on apricot quality during simulated transportation and subsequent cold storage, fruit quality parameters were measured at 0 h, after 48 h of simulated vibration, and on days 3, 6, and 9 of cold storage. The results showed that, compared with P2 and P3, P1 more effectively maintained fruit surface color and firmness, delayed declines in soluble solids content (SSC), titratable acidity (TA), ascorbic acid content, and moisture content, and reduced water loss and overall weight loss. P1 also suppressed the increase in respiration rate, enhanced peroxidase (POD) and catalase (CAT) activities, suppressed increases in polyphenol oxidase (PPO) activity and hydrogen peroxide (H_2_O_2_) accumulation, and reduced lipid peroxidation. Additionally, P1 alleviated damage to the cell wall, maintained the structural integrity of the pulp cell walls, and improved the percentage of sound fruit. Transmission electron microscopy (TEM) confirmed that P1 delayed the degradation of the pulp cell wall and maintained the structural integrity of fruit cells. In conclusion, P1 (plastic basket) was the optimal packaging method for maintaining postharvest quality of fresh apricots during simulated transportation and cold storage.

## 1. Introduction

Apricot (*Prunus armeniaca* L.), a member of the genus *Prunus* in the family Rosaceae, is characterized by bright color, unique flavor, and abundant nutrients (vitamins, minerals, dietary fiber, and bioactive compounds), and has high nutritional and commercial value [1]. As the center of origin and the leading producer of apricots, China ranks first in both cultivation area and yield. However, apricots have high postharvest respiration and transpiration rates; moreover, their ripening period coincides with the hot summer season, and they are highly susceptible to softening, decay, and quality deterioration, resulting in poor storability and transportability [2]. The underdevelopment of postharvest preservation technologies has resulted in high postharvest losses during circulation and has become a critical bottleneck restricting the high-quality development of the apricot industry. Therefore, developing effective strategies to maintain postharvest quality and extend shelf life is an urgent priority for both industry and academia.

From harvest to consumption, fruits undergo multiple logistical stages, including packaging, transportation, and cold storage. Among these stages, transportation is a key link connecting production areas and markets, and vibration during transport (caused by engine operation and road irregularities) can cause irreversible damage to fruit by disrupting cell membrane and cell wall integrity, accelerating respiration and water loss, and ultimately reducing fruit quality and commercial value [3]. Vibration stress exerts dual damaging effects on fruits, including direct physical damage manifested as epidermal abrasion, flesh bruising and tissue collapse, and indirect physiological damage that triggers a series of stress metabolic responses when vibration serves as an abiotic stress signal [4,5]. Studies have shown that simulated transportation vibration significantly accelerates quality deterioration in apricots [6], blueberries [7], grapes [8], and other fruits. Therefore, mitigating vibration-induced damage is essential for preserving postharvest fruit quality.

Packaging is one of the primary measures used to reduce damage caused by transport vibration [9]. It not only provides physical protection and cushioning, but also regulates the fruit microenvironment (via air permeability and heat dissipation), thereby inhibiting excessive respiration and oxidative damage and delaying senescence [10]. Common packaging materials for fruit include plastic baskets [11], corrugated cartons [12], and foam boxes [13], each of which has distinct advantages (e.g., plastic baskets with good air permeability and reusability, corrugated cartons with low cost and recyclability, foam boxes with excellent thermal insulation and cushioning). However, systematic studies on their protective effects on apricots under vibration stress, as well as the underlying physiological mechanisms, remain limited.

Postharvest quality changes are closely related to physiological and biochemical processes. The accumulation of reactive oxygen species (ROS) can cause lipid peroxidation and cellular damage [14]; PPO activation can trigger enzymatic browning [15]. At present, most studies focus on individual postharvest stages (e.g., only transport or only storage), and relevant analyses of fruit quality variations predominantly concentrate on commodity quality deterioration and physical damage [16], whereas research on the comprehensive effects of packaging on fruit quality, antioxidant capacity, and cell structure during both transportation and storage remains scarce—particularly with regard to ultrastructural changes in the cell wall.

To address these gaps, this study used fresh red apricots as experimental materials to investigate the effects of three commonly used packaging methods (plastic baskets, foam boxes, corrugated cartons) on fruit quality, physiological metabolism, and cell structure during simulated transport and cold storage. The findings are intended to provide theoretical support and practical guidance for optimizing apricot postharvest preservation technologies and promoting the high-quality development of the apricot industry.

## 2. Materials and Methods

### 2.1. Experimental Materials

Fresh red apricots were harvested from an orchard in the 8th Regiment, Alar City, Xinjiang Uyghur Autonomous Region, China. Fruits with uniform maturity (firmness: 8–9 N, soluble solids content (SSC): 17–18%) were selected. After harvest, the fruits were individually wrapped in thin foam mesh sleeves to prevent mechanical damage, packed into plastic baskets and immediately transported to a laboratory cold room by road. The fruits were precooled at 0–5 °C for 24 h before the experiment.

### 2.2. Experimental Treatment Methods

**Figure 1:**
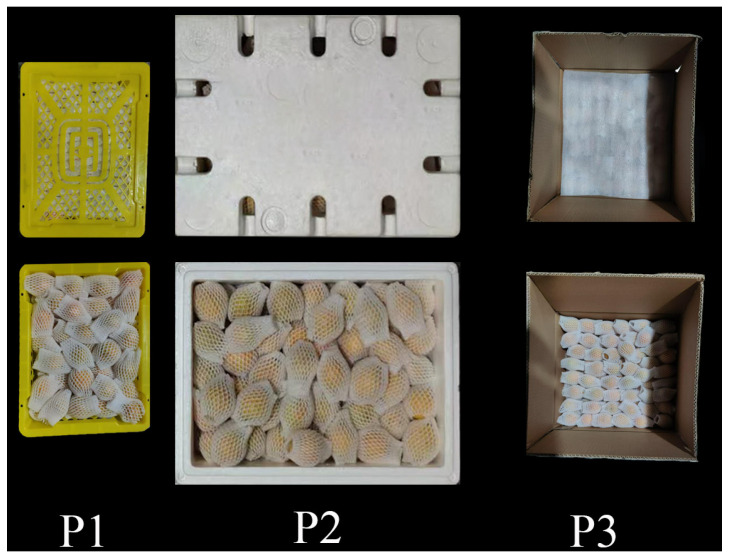
(**P1**) (plastic basket), (**P2**) (breathable foam box), and (**P3**) (perforated corrugated carton).

After precooling, fruits with uniform peel color, size, and shape and free from disease, insect damage, or mechanical injury were selected for packaging treatments. Three packaging treatments were established: P1 (plastic baskets, 28 cm × 21 cm × 11 cm, approximately 1.5 kg of fruit per basket); P2 (breathable foam boxes, 45.5 cm × 35.5 cm × 11.5 cm, with 10 ventilation holes on the top and bottom, approximately 1.8 kg of fruit per box); P3 (perforated corrugated cartons, 30.5 cm × 28.5 cm × 11 cm, with two small holes (1 cm in diameter) on each side, approximately 1.95 kg of fruit per carton) (Figure 1). All fruits were individually wrapped in thin pearl-cotton mesh sleeves before being placed into corresponding packages. Foam pads were placed at the bottom and top of the P1 and P3 packages to enhance cushioning, and absorbent paper was lined inside all three packages to absorb condensed water. After packaging, all samples were immediately placed in outer perforated corrugated cartons and placed on a simulated vehicle vibration table. The vibration frequency was set to 3 Hz, which is a typical value for trucks transporting stone fruits such as pears and plums [17], and simulated transportation vibration was applied for 48 h. After vibration treatment, all samples were transferred to a cold room at 0–5 °C for 9 d. Samples were collected at 0 h, after 48 h of simulated vibration, and on days 3, 6, and 9 of post-transport cold storage for the determination of various quality and physiological parameters.

### 2.3. Determination of Quality and Physiological Indicators

#### 2.3.1. Respiration Rate, Firmness, Soluble Solids Content and Surface Color

Respiration rate was determined using a fruit and vegetable respiration analyzer (JFQ-3150H, Beijing Junfang Physical and Chemical Technology Research Institute, Beijing, China). The respiration rate was measured using 20 fruits per replicate. Fruit firmness was determined using a texture analyzer (TMS-PILOT, Beijing Yingshenhengtai Technology Co., Ltd., Beijing, China) equipped with a P/5 cylindrical flat-headed probe. Three equidistant points in the equatorial region of each fruit were measured, with 20 fruits assessed per treatment, and the results were averaged. SSC was measured using a digital refractometer (PAL-1, Zhejiang Top Instrument Co., Ltd., Hangzhou, China). Color parameters (L*, a*, b*) were determined using a colorimeter (CR-400, Konica Minolta (China) Investment Co., Ltd., Shanghai, China). All measurements were performed in triplicate biological replicates to ensure data reliability.

#### 2.3.2. Sound Fruit Percentage, Weight Loss Percentage and Relative Electrical Conductivity

The sound fruit percentage was calculated as the ratio of undamaged fruits to the total number of fruits, expressed as a percentage. Formula:

$$P=\frac{N_{undamaged}}{N_{total}}\times 100\%$$

Weight loss percentage was calculated as the percentage reduction in fruit mass relative to the initial mass. Formula:

$$W=\frac{m_{initial-}m_{final}}{m_{initial}}\times 100\%$$

Relative electrical conductivity was determined using the conductivity method described by Cao et al. [18] with minor modifications. 2 g of uniform-sized flesh slices were obtained using a puncher and immersed in 20 mL of distilled water. After sealing and thorough shaking, the mixture was allowed to stand for 30 min, and the initial conductivity of the solution was recorded as C_1_. Subsequently, the solution was heated in a boiling water bath for 30 min. After cooling to room temperature, the final conductivity (C_2_) was determined. Formula:

$$REC=\frac{C_{1}}{C_{2}}\times 100\%$$

#### 2.3.3. Moisture Content, Ascorbic Acid, Titratable Acidity and Total Phenol Contents

Moisture content was determined using the direct drying method described by Kong et al. [19] with minor modifications. Flesh samples were weighed into pre-dried constant-weight weighing bottles and dried in an oven at 105 °C for 5 h. Subsequently, the bottles were transferred to a desiccator to cool for 30 min before reweighing. Results were expressed as a percentage on a fresh weight basis.

Ascorbic acid (Vc) content was determined using the 2,6-dichloroindophenol titration method described by Cao et al. [20] with minor modifications. 10 g of chopped flesh samples were homogenized with a small volume of 2% oxalic acid solution in a mortar (Oxalic acid, Sinopharm Chemical Reagent Co., Ltd., Shanghai, China). The homogenate was transferred to a 100 mL volumetric flask and diluted to the mark with 2% oxalic acid solution. After standing for 10 min, the mixture was filtered. Subsequently, 10 mL of filtrate was pipetted into an Erlenmeyer flask and titrated with standardized 2,6-dichloroindophenol sodium solution until a persistent pink color was maintained for 15 s (2,6-Dichlorophenolindophenol sodium salt, Shanghai Yuanye Bio-Technology Co., Ltd., Shanghai, China). Titrant consumption was recorded. A blank test was performed using 10 mL of 2% oxalic acid solution under identical conditions.

Titratable acidity (TA) was determined using the acid-base titration method described by Bai et al. [21] with minor modifications. 5 g of homogenized flesh samples were weighed into a beaker and transferred to a 100 mL volumetric flask using boiled and cooled distilled water. The solution was thoroughly shaken, diluted to the mark, and allowed to stand for 30 min for extraction before filtration. Exactly 10 mL of filtrate was pipetted into an Erlenmeyer flask, and three drops of 1% phenolphthalein indicator were added (Phenolphthalein, Sinopharm Chemical Reagent Co., Ltd., Shanghai, China). The mixture was titrated with standardized 0.01 mol/L sodium hydroxide solution until a pink hue persisted for 30 s (Sodium hydroxide, Sinopharm Chemical Reagent Co., Ltd., Shanghai, China). The volume of consumed titrant was recorded.

Total phenol content was determined using the Folin–Ciocalteu method described by Narra et al. [22] with minor modifications. One gram of flesh samples was extracted overnight with 5 mL of 80% ethanol at 4 °C (Anhydrous ethanol, Sinopharm Chemical Reagent Co., Ltd., Shanghai, China). The mixture was centrifuged at 12,000 rpm for 10 min at 4 °C to collect the supernatant. Subsequently, 0.125 mL of supernatant was mixed with 0.125 mL of Folin–Ciocalteu reagent and incubated in darkness for 6 min (Folin-Ciocalteu reagent, Beijing Solarbio Science & Technology Co., Ltd., Beijing, China). Then, 1.25 mL of 20% sodium carbonate solution and 1 mL of distilled water were added sequentially (Anhydrous sodium carbonate, Sinopharm Chemical Reagent Co., Ltd., Shanghai, China). The reaction system was incubated in the dark at room temperature for 1 h, and absorbance was measured at 760 nm using a spectrophotometer. A standard curve was established using gallic acid as the reference standard (Gallic acid, Sinopharm Chemical Reagent Co., Ltd., Shanghai, China).

#### 2.3.4. Catalase (CAT), Peroxidase (POD), Polyphenol Oxidase (PPO) Activities, MDA and Hydrogen Peroxide (H_2_O_2_) Contents

CAT, POD, and PPO activities, as well as malondialdehyde (MDA) and H_2_O_2_ contents, were determined according to the method of Cao et al. [20].

Catalase (CAT) activity was determined spectrophotometrically. Briefly, 5.0 g of flesh samples were mixed with 5.0 mL of extraction buffer (prepared with 0.1 mmol/L sodium phosphate buffer at pH 7.5, containing 5 mmol/L DTT and 5% PVP) and ground into a homogenate under ice bath conditions, then centrifuged at 12,000× *g* for 30 min at 4 °C (Dithiothreitol, Beijing Solarbio Science & Technology Co., Ltd., Beijing, China) (Polyvinylpyrrolidone, Sinopharm Chemical Reagent Co., Ltd., Shanghai, China). The supernatant was collected as the crude enzyme extract. The reaction mixture (3.0 mL) contained 2.9 mL of 20 mmol/L H_2_O_2_ (Prepared by dissolving a 30% hydrogen peroxide solution in a 50 mmol/L sodium phosphate buffer at pH 7.5) and 100 μL of enzyme extract (30% Hydrogen peroxide, Sinopharm Chemical Reagent Co., Ltd., Shanghai, China). Using distilled water as the blank, absorbance at 240 nm was recorded at 15 s (initial reading) and then every 30 s for 3 min.

Peroxidase (POD) activity was determined using the guaiacol colorimetric method (Guaiacol, Sinopharm Chemical Reagent Co., Ltd., Shanghai, China). Briefly, 5.0 g of flesh samples were mixed with 5.0 mL of extraction buffer (0.1 mol/L sodium acetate buffer, pH 5.5, containing 1 mmol/L PEG, 4% PVPP, and 1% Triton X-100) and ground into a homogenate under ice bath conditions (Polyethylene glycol 6000, Sinopharm Chemical Reagent Co., Ltd., Shanghai, China) (Polyvinylpolypyrrolidone, Beijing Solarbio Science & Technology Co., Ltd., Beijing, China) (Triton X-100, Beijing Solarbio Science & Technology Co., Ltd., Beijing, China). The homogenate was centrifuged at 12,000× *g* for 30 min at 4 °C, and the supernatant was collected as the crude enzyme extract. The reaction mixture contained 3.0 mL of 25 mmol/L guaiacol, 0.5 mL of enzyme extract, and 200 μL of 0.5 mol/L H_2_O_2_ (prepared in 50 mmol/L sodium acetate buffer, pH 5.5). Absorbance at 470 nm was recorded at 15 s and then every 1 min for 6 consecutive points.

Polyphenol oxidase (PPO) activity was determined spectrophotometrically. Briefly, 5.0 g of flesh samples were mixed with 5.0 mL of extraction buffer (0.1 mol/L sodium acetate, pH 5.5, containing 1 mmol/L PEG, 4% PVPP, and 1% Triton X-100) and ground into a homogenate under ice bath conditions. The homogenate was centrifuged at 12,000× *g* for 30 min at 4 °C, and the supernatant was collected as the crude enzyme extract (Catechol, Sinopharm Chemical Reagent Co., Ltd., Shanghai, China). The reaction mixture contained 4.0 mL of 50 mmol/L sodium acetate buffer (pH 5.5), 1.0 mL of 50 mmol/L catechol solution, and 100 μL of enzyme extract. Absorbance at 420 nm was recorded at 15 s (initial reading) and then every 1 min for 6 consecutive points.

Malondialdehyde (MDA) content was determined using the thiobarbituric acid (TBA) method (Thiobarbituric acid, Beijing Solarbio Science & Technology Co., Ltd., Beijing, China). Briefly, 1.0 g of flesh samples were homogenized in 5.0 mL of 100 g/L trichloroacetic acid (TCA) solution, then centrifuged at 10,000× *g* for 20 min at 4 °C (Trichloroacetic acid, Sinopharm Chemical Reagent Co., Ltd., Shanghai, China). The supernatant was collected for analysis. For the assay, 2.0 mL of supernatant was mixed with 2.0 mL of 6.7 g/L TBA (prepared in 0.05 mol/L NaOH), boiled for 20 min, cooled rapidly, and centrifuged again. Absorbance was measured at 450 nm, 532 nm, and 600 nm.

Hydrogen peroxide (H_2_O_2_) content was determined using the titanium sulfate method. For sample preparation, 5.0 g of flesh samples were mixed with 5.0 mL of pre-cooled acetone and ground into a homogenate under ice bath conditions, then centrifuged at 12,000× *g* for 20 min at 4 °C (Acetone, Sinopharm Chemical Reagent Co., Ltd., Shanghai, China). The supernatant was collected as the extract. For the assay, 1.0 mL of extract was mixed with 0.1 mL of 10% TiCl_4_-HCl solution and 0.2 mL of concentrated ammonia, then centrifuged (Titanium tetrachloride, Sinopharm Chemical Reagent Co., Ltd., Shanghai, China) (Hydrochloric acid, Sinopharm Chemical Reagent Co., Ltd., Shanghai, China) (Concentrated ammonia water, Sinopharm Chemical Reagent Co., Ltd., Shanghai, China). The precipitate was washed three times with acetone, dissolved in 3.0 mL of 2 mol/L H_2_SO_4_, and the absorbance was measured at 412 nm. H_2_O_2_ content was calculated from a standard curve prepared with known H_2_O_2_ concentrations and expressed as μmol/g fresh weight.

#### 2.3.5. Ultrastructural Observation of Pulp Cell Walls

Pulp samples for transmission electron microscopy (TEM) were prepared with minor modifications based on the method of Fan et al. [23]. Briefly, pulp samples were cut into 1 mm^3^ cubes, rinsed three times for 15 min each with 0.1 mol/L phosphate buffer (pH 7.0), and then fixed in 1% osmium tetroxide solution for 2 h. After fixation, the samples were rinsed three additional times with the same phosphate buffer. Samples were then dehydrated in a graded ethanol series (30%, 50%, 70%, 80%, 90%, 95%) for 15 min per step, immersed in 100% ethanol for 20 min, and finally soaked in pure acetone for 20 min. Subsequently, samples were infiltrated with an embedding resin–acetone mixture (*v*/*v* = 1:1) for 1 h, then transferred to a mixture of embedding resin and acetone (*v*/*v* = 3:1) for 3 h, and finally embedded in pure embedding resin overnight. The embedded samples were transferred to embedding capsules and polymerized at 70 °C overnight. Ultrathin sections (80 nm thick) were cut using an ultramicrotome and then stained with 50% ethanol-saturated uranyl acetate and lead citrate for 10 min each. After air-drying, sections were examined under a transmission electron microscope (JEOL JEM-F200, JEOL Ltd., Tokyo, Japan).

### 2.4. Data Analysis and Statistical Methods

Raw data were compiled and processed using Microsoft Excel 2019 (Microsoft Corporation, Redmond, WA, USA). Statistical analysis was performed using SPSS 27.0 software (IBM Corporation, Armonk, NY, USA), and figures were generated using Origin 2024 software (OriginLab Corporation, Northampton, MA, USA). All error bars indicate the standard deviation (SD) of three independent biological replicates. Different lowercase letters at the same sampling time indicate significant differences among treatments (*p* < 0.05) by Duncan’s multiple range test.

## 3. Results

### 3.1. Effects of Packaging Methods on Sound Fruit Percentage, Weight Loss Percentage, and Moisture Content of Fresh Apricots During Simulated Transportation and Storage

**Figure 2:**
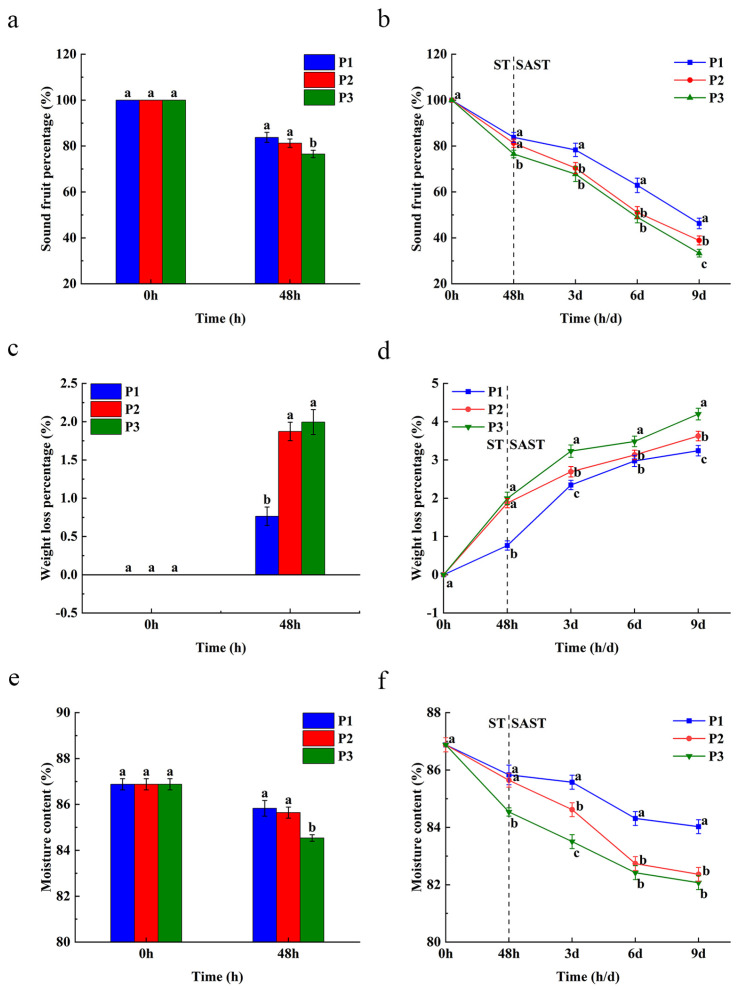
Effects of packaging methods on sound fruit percentage, weight loss percentage and moisture content of fresh red apricots during simulated transportation and storage (Panels (**a**,**b**): sound fruit percentage; Panels (**c**,**d**): weight loss percentage; Panels (**e**,**f**): moisture content. In panels (**b**,**d**,**f**): ST: simulated transportation; SAST: storage after simulated transportation; different lowercase letters indicate significant differences at *p* < 0.05 among treatments, and this abbreviation convention applies to all subsequent figures).

During simulated transportation, the sound fruit percentage decreased in all treatments. After 48 h of vibration, the sound fruit percentages of P1 and P2 were 83.77% and 81.26%, respectively, which were significantly higher than that in P3 (76.54%) (*p* < 0.05) (Figure 2a). After 48 h of simulated transportation, weight loss increased in all groups, with P1 showing the lowest weight loss (0.76%) (*p* < 0.05) (Figure 2c). Meanwhile, the moisture content decreased in all groups, and P1 and P2 retained significantly higher moisture contents (85.83% and 85.64%, respectively) than P3 (84.54%) (Figure 2e).

During the post-transport storage period, the sound fruit percentage in all packaging treatments gradually decreased with storage time. On day 9 of cold storage, the sound fruit percentage of P1 was 46.30%, which was 7.42% and 12.96% higher than the values in P2 and P3, respectively (*p* < 0.05) (Figure 2b). Weight loss increased continuously throughout the storage period. On day 9 of storage, the weight loss percentage of P1 was 3.24% (*p* < 0.05) (Figure 2d). Correspondingly, the moisture content of P1 decreased to 84.02%, which was still significantly higher than the values in P2 (82.36%) and P3 (82.07%) (*p* < 0.05) (Figure 2f).

### 3.2. Effects of Packaging Methods on Respiration Rate, SSC, TA, and Firmness of Fresh Apricots During Simulated Transportation and Storage

**Figure 3:**
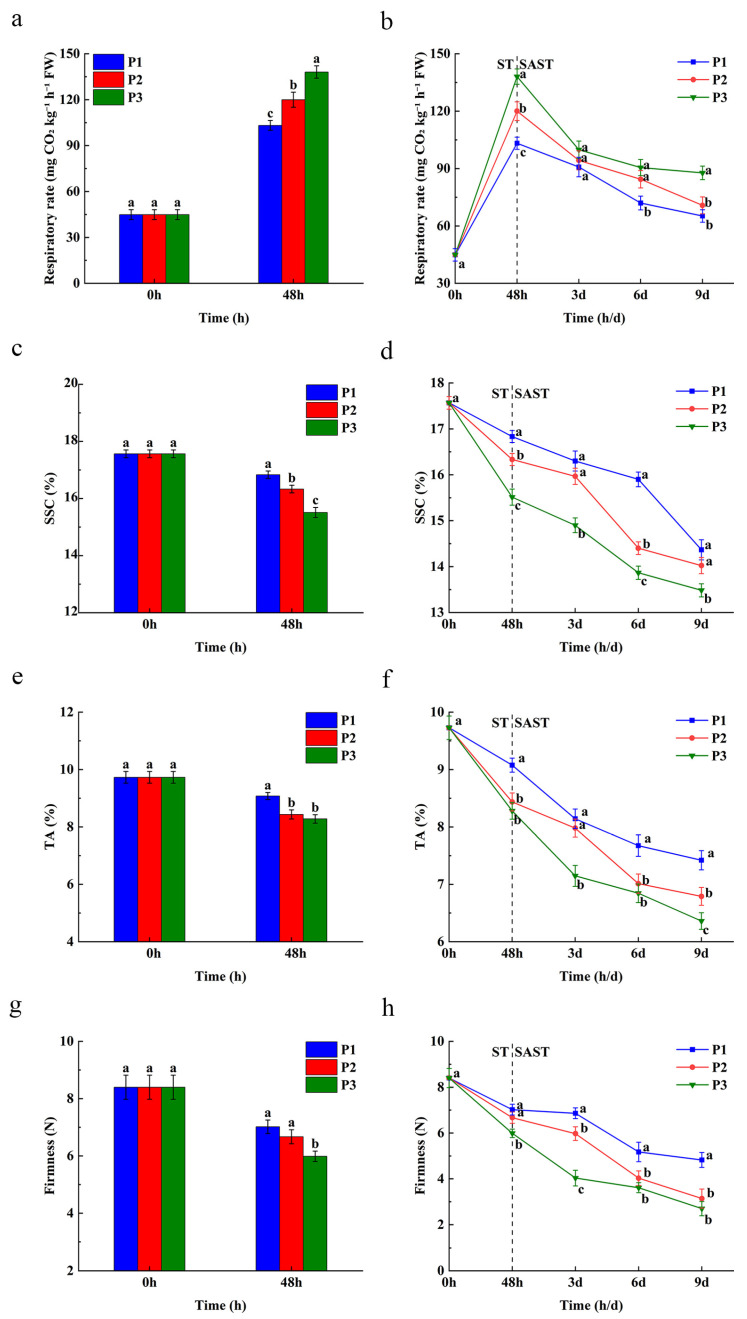
Effects of packaging methods on respiration rate, soluble solids content, titratable acidity content and firmness of fresh red apricots during simulated transportation and storage (Panels (**a**,**b**): respiration rate; Panels (**c**,**d**): soluble solids content; Panels (**e**,**f**): titratable acidity content; Panels (**g**,**h**): firmness; different lowercase letters indicate significant differences at *p* < 0.05 among treatments).

During simulated transport, the respiration rate increased in all treatments. After 48 h of vibration, the respiration rate of P1 (103.23 mgCO_2_·kg^−1^·h^−1^) was significantly lower than that of P2 (120.00 mgCO_2_·kg^−1^·h^−1^) and P3 (138.09 mgCO_2_·kg^−1^·h^−1^), by 14% and 25%, respectively (*p* < 0.05) (Figure 3a). After 48 h of simulated transportation, SSC and TA slightly decreased in all groups. P1 showed an SSC of 16.83% and a TA of 9.08%, both of which were significantly higher than those of P2 (16.33%, 8.44%) and P3 (15.51%, 8.28%) (*p* < 0.05) (Figure 3c,e). After 48 h of simulated transportation, P1 and P2 had firmness of 7.02 N and 6.67 N, respectively, while P3 dropped to 5.99 N. The firmness values of P1 and P2 were 17% and 12% higher than those of P3, respectively (*p* < 0.05) (Figure 3g).

On day 9 of storage, the respiration rates of P1 and P2 were 65.21 and 70.76 mgCO_2_·kg^−1^·h^−1^, respectively, which were significantly lower than that of P3 (87.75 mgCO_2_·kg^−1^·h^−1^) by 26% and 19%, respectively (*p* < 0.05) (Figure 3b). With prolonged storage, SSC and TA continued to decrease. By day 9, P1 and P2 SSC were 14.37% and 14.02%, significantly higher than P3 (13.48%) (*p* < 0.05) (Figure 3d). P1 TA was 7.42%, significantly higher than P2 (6.79%) and P3 (6.36%) (*p* < 0.05) (Figure 3f). Fruit firmness declined markedly during storage. By day 9, P1 firmness remained at 4.83 N, 54% and 79% higher than P2 and P3, respectively (*p* < 0.05) (Figure 3h).

### 3.3. Effects of Packaging Methods on Color of Fresh Apricots During Simulated Transportation and Storage

**Figure 4:**
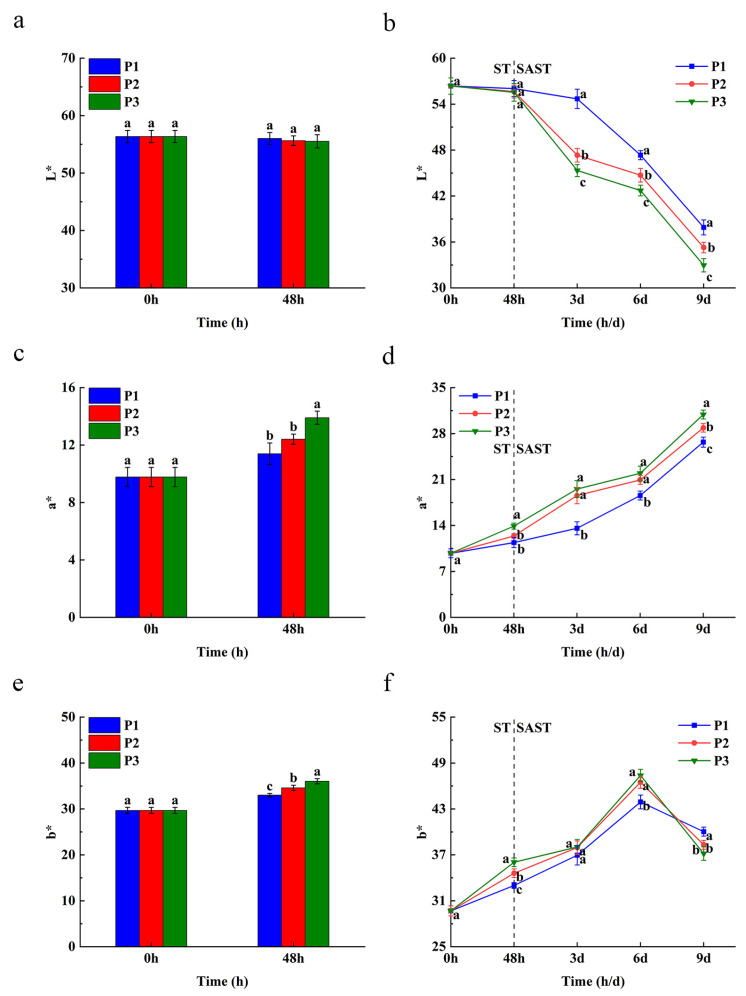
Effects of packaging methods on color parameters of fresh red apricots during simulated transportation and storage (Panels (**a**,**b**): L* value; Panels (**c**,**d**): a* value; Panels (**e**,**f**): b* value; different lowercase letters indicate significant differences at *p* < 0.05 among treatments).

During simulated transportation, L* values declined in all groups. After 48 h of vibration, L* values of P1, P2 and P3 were 56.03, 55.64 and 55.53, respectively, with no significant differences among treatments (Figure 4a). Meanwhile, a* and b* values increased in all groups. At 48 h, the a* values of P1 (11.40) and P2 (12.41) were significantly lower than that of P3 (13.91) by 18% and 11%, respectively (*p* < 0.05) (Figure 4c). b* values of P1, P2 and P3 were 33.00, 34.60 and 36.03, respectively; P1 b* value was 5% and 8% lower than P2 and P3 (*p* < 0.05) (Figure 4e).

During post-transport cold storage, changes in color parameters became more pronounced, with obvious differences among groups. L* values continued to decline with storage time. On day 9, L* values of P1, P2 and P3 were 37.90, 35.27 and 32.95, respectively; P1 L* value was 7% and 15% higher than P2 and P3 (*p* < 0.05) (Figure 4b). On day 9, P1 a* value (26.70) was significantly lower than P2 (28.88) and P3 (30.91) (*p* < 0.05) (Figure 4d). b* values in all groups rose first and then fell, peaking on day 6. On day 9, P1 b* value (40.03) was significantly higher than P2 (38.32) and P3 (37.17) (*p* < 0.05) (Figure 4f).

### 3.4. Effects of Packaging Methods on Ascorbic Acid Content, Total Phenol Content, and PPO Activity of Fresh Apricots During Simulated Transportation and Storage

**Figure 5:**
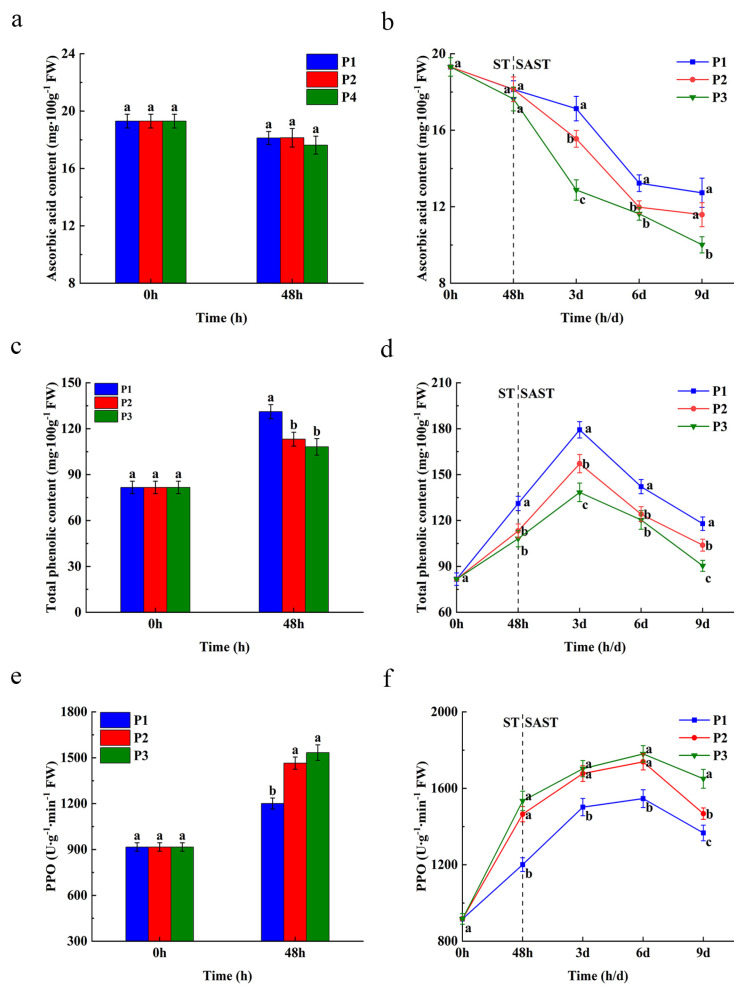
Effects of packaging methods on ascorbic acid content, total phenol content and PPO activity of fresh red apricots during simulated transportation and storage (Panels (**a**,**b**): ascorbic acid content; Panels (**c**,**d**): total phenol content; Panels (**e**,**f**): PPO activity; different lowercase letters indicate significant differences at *p* < 0.05 among treatments).

During simulated transportation, ascorbic acid contents decreased in all packaging groups. After 48 h of vibration treatment, ascorbic acid contents of P1, P2 and P3 were 18.13, 18.14 and 17.64 mg·100 g^−1^, respectively, with no significant differences among treatments (Figure 5a). This indicated that transportation vibration had no significant differential effect on ascorbic acid content among the three packaging groups at this stage. Total phenol contents increased in all groups during transportation. The total phenol content of P1 reached 131.15 mg·100 g^−1^ (*p* < 0.05) (Figure 5c), 16% and 21% higher than P2 (113.18 mg·100 g^−1^) and P3 (108.16 mg·100 g^−1^), respectively. PPO activity increased in all groups during transport. After 48 h, PPO activity of P1 was 1200.81 U·g^−1^·min^−1^ (*p* < 0.05) (Figure 5e), 18% and 22% lower than P2 (1464.85 U·g^−1^·min^−1^) and P3 (1534.06 U·g^−1^·min^−1^).

During post-transport cold storage, ascorbic acid content continued to decline in all groups. On day 9, ascorbic acid contents of P1 and P2 were 12.73 mg·100 g^−1^ and 11.59 mg·100 g^−1^, respectively (*p* < 0.05) (Figure 5b), 27% and 16% higher than P3 (10.01 mg·100 g^−1^). Total phenol content first increased and then decreased during storage, peaking on day 3. The peak total phenol content of P1 was 179.28 mg·100 g^−1^ (*p* < 0.05), 14% and 30% higher than P2 (157.17 mg·100 g^−1^) and P3 (138.41 mg·100 g^−1^), respectively. By day 9, P1 total phenol content remained at 117.92 mg·100 g^−1^ (*p* < 0.05) (Figure 5d), 13% and 30% higher than P2 (103.90 mg·100 g^−1^) and P3 (90.37 mg·100 g^−1^), respectively. PPO activity also increased first and then decreased during storage, peaking on day 6. The peak PPO activity of P1 was 1546.24 U·g^−1^·min^−1^ (*p* < 0.05), 11% and 13% lower than P2 (1738.88 U·g^−1^·min^−1^) and P3 (1780.22 U·g^−1^·min^−1^), respectively. By day 9, P1 PPO activity was 1366.60 U·g^−1^·min^−1^ (*p* < 0.05) (Figure 5f), 7% and 17% lower than P2 (1467.62 U·g^−1^·min^−1^) and P3 (1650.14 U·g^−1^·min^−1^), respectively.

### 3.5. Effects of Packaging Methods on Relative Electrical Conductivity and MDA Content of Fresh Apricots During Simulated Transportation and Storage

**Figure 6:**
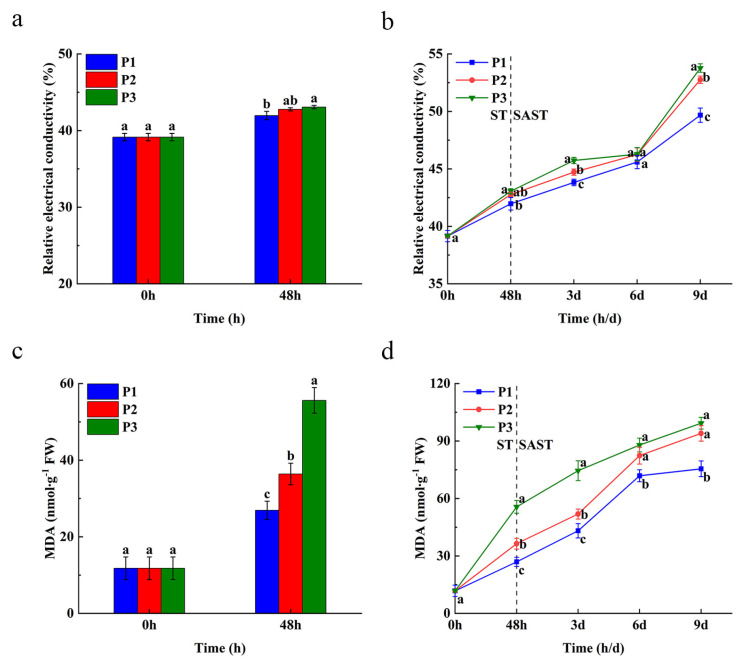
Effects of packaging methods on relative electrical conductivity and MDA content of fresh red apricots during simulated transportation and storage (Panels (**a**,**b**): relative electrical conductivity; Panels (**c**,**d**): MDA content; different lowercase letters indicate significant differences at *p* < 0.05 among treatments).

During simulated transportation, relative electrical conductivity increased in all packaging groups. After 48 h of vibration treatment, relative electrical conductivities of P1, P2 and P3 were 41.97%, 42.77% and 43.66%, respectively, and P1 was significantly lower than P3 (*p* < 0.05) (Figure 6a). MDA contents increased in all groups during transportation. After 48 h, the MDA content of P1 was 26.91 nmol·g^−1^ (*p* < 0.05) (Figure 6c), 26% and 52% lower than P2 (36.39 nmol·g^−1^) and P3 (55.61 nmol·g^−1^), respectively.

On day 9, the relative electrical conductivity of P1 was 49.67% (*p* < 0.05) (Figure 6b), significantly lower than P2 (52.76%) and P3 (53.77%). MDA contents increased continuously in all packaging treatments. On day 9, the MDA content of P1 was 75.49 nmol·g^−1^ (*p* < 0.05) (Figure 6d), 20% and 24% lower than P2 (93.97 nmol·g^−1^) and P3 (99.31 nmol·g^−1^), respectively.

### 3.6. Effects of Packaging Methods on POD Activity, CAT Activity, and H_2_O_2_ Content of Fresh Apricots During Simulated Transportation and Storage

**Figure 7:**
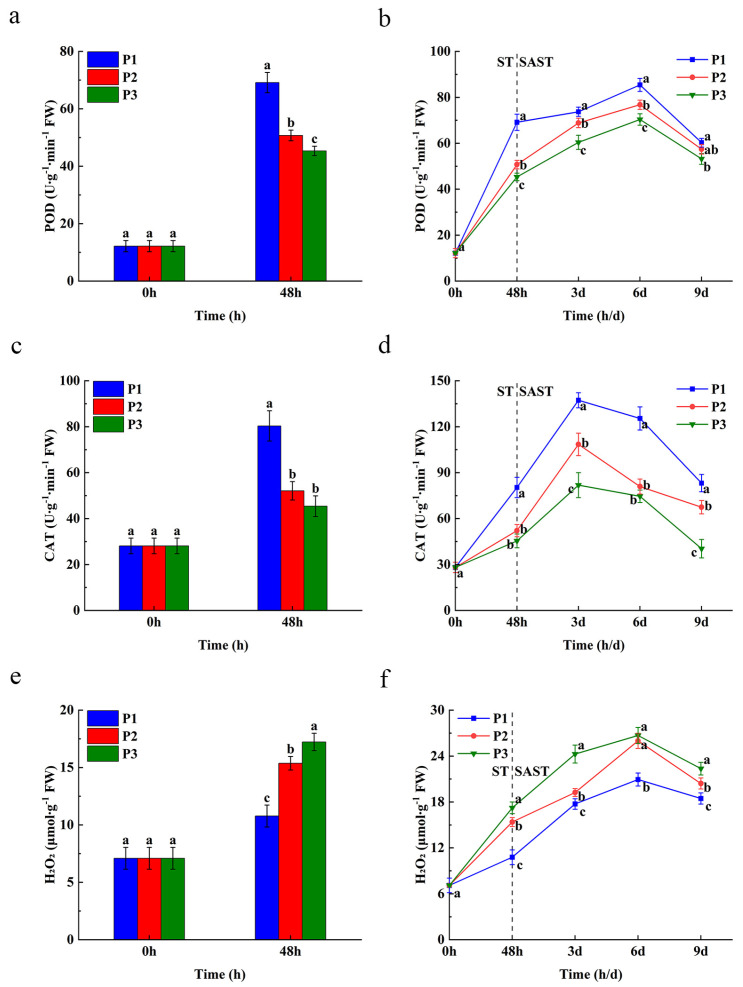
Effects of packaging methods on POD activity, CAT activity and H_2_O_2_ content of fresh red apricots during simulated transportation and storage (Panels (**a**,**b**): POD activity; Panels (**c**,**d**): CAT activity; Panels (**e**,**f**): H_2_O_2_ content; different lowercase letters indicate significant differences at *p* < 0.05 among treatments).

Under simulated transportation vibration, POD and CAT activities increased in all treatments. After 48 h, POD activity in P1 reached 69.16 U·g^−1^·min^−1^ (*p* < 0.05) (Figure 7a), 36% and 53% higher than P2 (50.74 U·g^−1^·min^−1^) and P3 (45.35 U·g^−1^·min^−1^), respectively. CAT activity in P1 was 80.35 U·g^−1^·min^−1^ (*p* < 0.05) (Figure 7c), 54% and 77% higher than P2 (52.13 U·g^−1^·min^−1^) and P3 (45.42 U·g^−1^·min^−1^), respectively. H_2_O_2_ content increased markedly in all groups during simulated transportation. After 48 h, H_2_O_2_ content in P1 was 10.78 μmol·g^−1^ (*p* < 0.05) (Figure 7e), 30% and 37% lower than P2 (15.38 μmol·g^−1^) and P3 (17.23 μmol·g^−1^), respectively.

During post-transport cold storage, POD and CAT activities in all groups first increased and then decreased. POD activity peaked on day 6, and CAT activity peaked on day 3. At the POD peak, P1 activity reached 85.41 U·g^−1^·min^−1^ (*p* < 0.05), 11% and 21% higher than P2 (76.78 U·g^−1^·min^−1^) and P3 (70.36 U·g^−1^·min^−1^), respectively. On day 9, P1 POD activity remained at 60.27 U·g^−1^·min^−1^ (*p* < 0.05) (Figure 7b), significantly higher than P3 (53.19 U·g^−1^·min^−1^). At the CAT peak, P1 activity reached 137.28 U·g^−1^·min^−1^ (*p* < 0.05), 27% and 68% higher than P2 (108.47 U·g^−1^·min^−1^) and P3 (81.86 U·g^−1^·min^−1^), respectively. By day 9, P1 CAT activity was 83.15 U·g^−1^·min^−1^ (*p* < 0.05) (Figure 7d), 23% and 106% higher than P2 and P3. H_2_O_2_ content in all groups also peaked on day 6, then decreased. At the peak, P1 H_2_O_2_ content was 20.93 μmol·g^−1^ (*p* < 0.05), significantly lower than P2 (25.99 μmol·g^−1^) and P3 (26.69 μmol·g^−1^). On day 9, P1 H_2_O_2_ content was 18.46 μmol·g^−1^ (*p* < 0.05) (Figure 7f), 9% and 17% lower than P2 (20.42 μmol·g^−1^) and P3 (22.36 μmol·g^−1^).

### 3.7. Correlation Analysis Between Indicators During Simulated Transportation and Storage

**Figure 8:**
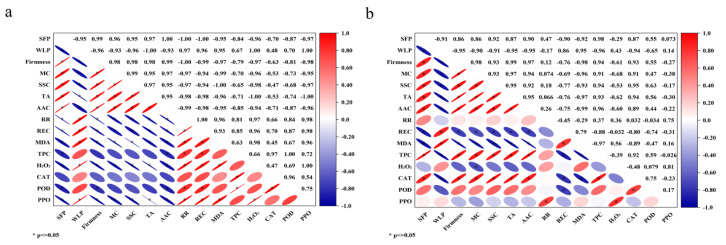
Correlation Analysis among various indicators during simulated transportation and storage (Panel (**a**): simulated transportation stage; Panel (**b**): post-transport cold storage stage; * indicates significant correlation at *p* < 0.05).

During simulated transportation, strong correlations were observed among the various indicators, indicating close relationships between physiological metabolism and quality changes (Figure 8a). Sound fruit percentage was significantly negatively correlated with weight loss percentage (r = −0.95), and significantly positively correlated with firmness, moisture content, SSC, TA, and Vc content (r = 0.99, 0.96, 0.95, 0.97, 1.00, respectively). It was also significantly negatively correlated with respiration rate, relative electrical conductivity, MDA content, and PPO activity (r = −1.00, −1.00, −0.95, −0.97, respectively). These results showed that lower weight loss, better texture and nutrient retention, and weaker respiration, membrane damage, and enzymatic browning corresponded to a higher sound fruit percentage during transportation. Weight loss percentage was significantly negatively correlated with firmness, moisture content, SSC, TA and Vc content (r = −0.96, −0.93, −0.96, −1.00, −0.93, respectively), and significantly positively correlated with respiration rate, relative electrical conductivity, MDA content, H_2_O_2_ content and PPO activity (r = 0.97, 0.96, 0.95, 1.00, 1.00, respectively). This indicated that water loss was a core driver of apricot quality deterioration during transportation, and excessive weight loss induced cellular dehydration, a respiratory burst, lipid peroxidation, and enzymatic browning. Total phenol content was significantly positively correlated with CAT and POD activities (r = 0.97, 1.00, respectively), and moderately positively correlated with MDA and H_2_O_2_ contents (r = 0.63, 0.66, respectively), suggesting that phenolic compounds acted synergistically with antioxidant enzymes to scavenge reactive oxygen species, inhibit lipid peroxidation, and delay cell senescence and damage.

During post-transport cold storage, the strength and direction of correlations among indicators changed notably, reflecting the complex physiological regulation that occurs during fruit senescence (Figure 8b). Sound fruit percentage remained significantly negatively correlated with weight loss percentage, relative electrical conductivity and MDA content (r = −0.91, −0.90, −0.92, respectively), and significantly positively correlated with firmness, moisture content, SSC, TA, ascorbic acid content and total phenol content (r = 0.86, 0.86, 0.92, 0.87, 0.90, 0.98, respectively). The overall strength of the correlations was lower than that observed during transportation, indicating that quality deterioration during storage was driven by more diverse factors rather than by a single dominant factor. Weight loss percentage remained extremely significantly negatively correlated with firmness, moisture content, SSC, TA, ascorbic acid content and total phenol content (r = −0.95, −0.90, −0.91, −0.95, −0.95, −0.96, respectively), weakly negatively correlated with respiration rate (r = −0.17), and significantly positively correlated with relative electrical conductivity and MDA content (r = 0.86, 0.95, respectively). This suggested that water loss remained a basic inducer of quality deterioration in storage, whereas the direct contribution of respiratory metabolism to membrane damage was greatly weakened. Total phenol content was significantly positively correlated with CAT and POD activities (r = 0.92, 0.59, respectively), and significantly negatively correlated with MDA and H_2_O_2_ contents (r = −0.97, −0.39, respectively), with weaker correlations than during transportation. This indicated that the antioxidant defense capacity of the fruit declined with senescence, weakening the synergistic ROS-scavenging effect of phenolics and antioxidant enzymes. Respiration rate was significantly positively correlated with PPO activity (r = 0.75), suggesting that enzymatic browning became a key driver of appearance deterioration during storage.

### 3.8. Effects of Packaging Methods on the Ultrastructure of Pulp Cell Walls of Fresh Apricots During Simulated Transportation and Storage

**Figure 9:**
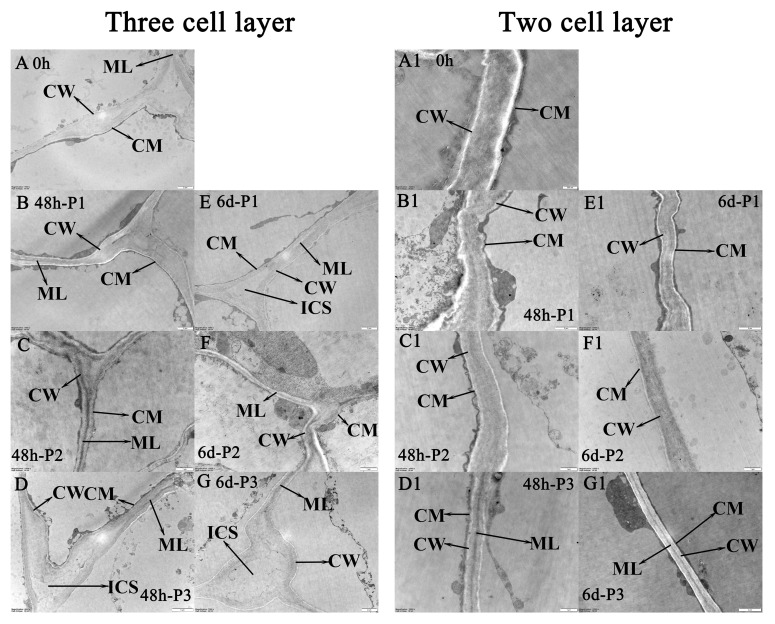
Effects of different packaging methods on the ultrastructure of flesh cell walls in fresh red apricots during simulated transportation and storage (CW: cell wall, CM: cell membrane, ML: middle lamella, ICS: intercellular space; Panel (**A**,**A1**): samples at 0 h of simulated transportation; magnification: Panel (**D**): ×4000; Panels (**A**,**B**,**E**,**G**): ×7000; Panels (**B1**,**E1**,**G1**): ×10,000; Panels (**C**,**F**,**C1**,**D1**,**F1**): ×15,000; Panel (**A1**): ×25,000).

At the initial stage of simulated transportation, the pulp cell wall structure of fresh apricots remained intact. The cell membrane adhered tightly to the cell wall and exhibited a clear bilayer structure, and the intercellular middle lamella was smooth with distinct boundaries (Figure 9A,A1). After 48 h of vibration, the cell wall structures in all groups showed varying degrees of alteration. In P1, the triangular regions of the cells and the bilayer structure between adjacent cells remained relatively intact (Figure 9B,B1). The cell wall thickness was uniform with only slight thinning and degradation; the cell membrane remained smooth and tightly attached to the cell wall, with only minor local detachment; the middle lamella was clear and smooth, with no obvious intercellular spaces or plasmolysis in the triangular regions. By contrast, more severe cell wall damage was observed in P2 (Figure 9C,C1) and P3 (Figure 9D,D1). These results indicated that P1 effectively alleviated cell wall degradation and better maintained structural integrity during simulated transportation.

On day 6 of post-transport cold storage, the pulp cell walls in all three packaging groups showed thinning and degradation, with obvious differences among groups. Cell wall degradation in P1 (Figure 9E,E1) was significantly milder than in P2 (Figure 9F,F1) and P3 (Figure 9G,G1). P1 exhibited only a slight reduction in cell wall thickness and uniform morphology in the triangular regions after degradation; the cell membrane structure remained relatively intact, with a clear middle lamella; the intercellular spaces in the triangular regions were small, and the bilayer structure between adjacent cells was thicker and more complete than in P2 and P3. These results were consistent with those observed during transportation, confirming that P1 packaging continued to protect the structural integrity of apricot pulp cell walls and delay degradation during subsequent cold storage.

## 4. Discussion

Fresh apricots exhibit vigorous postharvest respiration and transpiration. Ripening during high-temperature seasons leads to rapid quality deterioration, including water loss, wilting, softening, enzymatic browning and decay. The main causes include excessive respiration, rapid water loss, cell membrane damage, and enhanced oxidative stress [24]. As an important physical postharvest measure, packaging regulates the fruit microenvironment, reduces water loss, relieves mechanical vibration injury, and suppresses respiration and oxidative reactions, thereby delaying senescence [25]. This study investigated the effects of plastic baskets (P1), foam boxes (P2) and corrugated cartons (P3) on apricot quality and physiology during simulated transportation and storage. The results showed that P1 had significantly better preservation efficacy, which was related to its material properties, air permeability, cushioning effect, and heat dissipation capacity.

Respiration rate is a key physiological indicator characterizing postharvest respiratory metabolic activity of fruits. Elevated respiration rate accelerates endogenous nutrient consumption, promotes fruit senescence, and ultimately shortens the storage and preservation period [26]. The results demonstrated that P1 treatment significantly inhibited respiration rate elevation in red apricots, effectively reduced the respiratory metabolic peak, and maintained consistently low respiratory levels throughout late storage (Figure 3b). Respiratory metabolism is the dominant physiological process driving postharvest fruit senescence and deterioration. Excessively high respiration rate continuously depletes nutritional substrates, including soluble solids and organic acids, thereby exacerbating fruit quality degradation and accelerating the senescence process [27]. Soluble solids content (SSC) and titratable acidity (TA), which reflect fruit sugar and organic acid accumulation, respectively, are critical quality parameters determining the flavor and taste profile of red apricots [28]. Experimental findings revealed that P1 treatment markedly slowed the decline rates of SSC and TA contents, effectively preserving flavor compounds (Figure 3d,f). Regarding the underlying mechanism, it is hypothesized that the excellent breathability of plastic crates facilitates efficient gas exchange between fruits and the surrounding environment, which in turn suppresses the activity of respiratory metabolism-related enzymes and minimizes respiratory nutrient loss. This conclusion is consistent with the findings of Du et al. [29], who optimized the fruit storage microenvironment by developing breathable tunable composite preservation films and similarly delayed browning and retarded senescence in fruits and vegetables.

Water loss and wilting are the most common quality deterioration problems during fruit and vegetable storage and transportation. Excessive fruit water loss causes epidermal luster loss and flesh shrinkage, significantly reducing commercial value [30]. Weight loss rate is a core indicator for evaluating postharvest preservation efficacy and commercial quality of fruits and vegetables. Postharvest weight loss primarily arises from transpiration-induced water dissipation, accompanied by respiratory metabolism-derived dry matter consumption. Excessively high weight loss rate exacerbates fruit wilting and shrinkage, severely impairing appearance quality and marketability [31]. Water is the fundamental substance maintaining cell turgor pressure and crisp-tender texture of fruits and vegetables. Continuous decline in moisture content directly induces flesh tissue deterioration, making it a key physiological parameter for measuring fruit and vegetable freshness [32]. The results demonstrated that red apricots in the P1 treatment group exhibited significantly lower weight loss rate than those in the P2 and P3 groups, and P1 treatment sustained higher fruit moisture content throughout storage (Figure 2d,f). This indicates that P1 packaging possesses superior moisture retention and preservation performance compared with the other two treatments, effectively reducing fruit transpiration water loss. Regarding the underlying mechanism, P1 packaging exhibits excellent heat dissipation and breathability, which rapidly dissipates the field heat of red apricots and the respiratory heat generated during storage. This effectively alleviates heat accumulation-induced rapid water evaporation, maintains fruit cell water balance, and consequently inhibits fruit water loss and wilting. This finding is consistent with Dong et al. [33], who optimized the storage microenvironment by constructing a double-layer humidity-regulating packaging system, effectively reducing postharvest water loss in strawberries and significantly improving their preservation effect.

Color is the most intuitive and core indicator of fruit and vegetable appearance quality [34]. Postharvest color deterioration during storage and transportation is mainly attributed to enzymatic browning and pigment oxidative degradation [35,36]. Among these, elevated polyphenol oxidase (PPO) activity is the key trigger for fruit browning, causing abnormal color difference fluctuations, severe appearance quality damage, and reduced commercial value [35]. The results demonstrated that L* values of red apricots in the P1 group were significantly higher than those in the P2 and P3 groups, while a* and b* values showed more moderate variation during storage (Figure 4b,d,f). Additionally, P1 treatment effectively inhibited PPO activity and maintained consistently low enzyme levels throughout storage (Figure 5f). These findings indicate that P1 packaging effectively maintains red apricot color stability during storage, preserves color parameters close to initial values, and significantly reduces browning severity. Vitamin C (Vc) and total phenols are key indicators of fruit nutritional quality and antioxidant capacity. As a critical endogenous antioxidant, Vc not only determines nutritional quality but also delays oxidative senescence by scavenging reactive oxygen species (ROS) [37]. Phenolic compounds, important secondary metabolites of fruits and vegetables, exhibit strong antioxidant activity, and their content changes directly reflect fruit stress resistance and senescence degree [38]. Stable retention of both compounds effectively inhibits oxidative browning and maintains postharvest quality stability. In this experiment, red apricots in the P1 group consistently maintained higher Vc and total phenol contents throughout storage (Figure 5b,d), indicating that this packaging effectively preserves red apricot antioxidant capacity and alleviates oxidative damage. Fruit firmness is a critical quality parameter determining commercial value and storability. Excessive postharvest softening significantly increases the risk of mechanical damage and pathogen infection, accelerating quality deterioration [39]. The results showed that red apricots in the P1 group maintained higher firmness throughout the storage cycle (Figure 3h), indicating that P1 packaging effectively buffers mechanical stress during transportation, reduces texture damage from vibration and extrusion, and sustains fruit texture stability. It should be noted that Package 1 contained the lowest fruit weight, which reduced the mechanical stress caused by gravity and thereby alleviated vibration damage to the fruits [40]. Taken together, results from color, antioxidant, and texture indicators demonstrate that P1 packaging significantly inhibits postharvest enzymatic browning of red apricots, delays color deterioration, and effectively maintains both external appearance and internal quality. Regarding the underlying mechanism, P1 packaging exhibits excellent cushioning and protective properties, significantly reducing mechanical damage from simulated transportation vibration, thereby minimizing cell rupture and PPO activation at the source. Meanwhile, by maintaining high Vc and total phenol levels, it enhances fruit endogenous antioxidant capacity, effectively inhibits oxidative degradation of pigments such as carotenoids and anthocyanins, and ultimately stabilizes color quality. This finding is consistent with Feng et al. [41], who reported that cushioning packaging effectively delays postharvest browning and maintains quality in soft fruits.

Cell membrane damage and oxidative stress imbalance are the core physiological mechanisms driving postharvest fruit senescence and deterioration. Malondialdehyde (MDA), a characteristic metabolite of membrane lipid peroxidation, directly reflects the degree of oxidative damage to fruit cell membranes [42]. Hydrogen peroxide (H_2_O_2_) is the primary reactive oxygen species (ROS) in plants. Its excessive accumulation exacerbates membrane oxidative damage, disrupts structural integrity, and increases cell membrane permeability and fruit relative electrical conductivity [43,44]. Peroxidase (POD) and catalase (CAT) are the core endogenous antioxidant enzymes in fruits, which efficiently scavenge excess accumulated ROS in vivo, maintain cellular oxidative metabolic homeostasis, and serve as key physiological indicators of fruit stress resistance and anti-aging capacity [45]. The results demonstrated that MDA and H_2_O_2_ contents, as well as relative electrical conductivity, of red apricots in the P1 group were significantly lower than those in the P2 and P3 groups during storage, while POD and CAT activities were significantly higher (Figure 6 and Figure 7). These findings confirm that P1 packaging effectively inhibits membrane lipid peroxidation in red apricots, reduces excess ROS accumulation, and significantly enhances fruit antioxidant enzyme activities. By strengthening the endogenous antioxidant system capacity, it alleviates cell membrane structural damage and ultimately delays postharvest fruit senescence. Regarding the underlying mechanism, P1 packaging effectively inhibits excessive respiratory metabolism, reduces water loss, and stabilizes fruit color quality by regulating the storage and transportation microenvironments. A favorable microenvironment positively regulates the expression of antioxidant enzyme-related genes, continuously enhancing postharvest fruit stress resistance and antioxidant capacity. This finding is consistent with Li et al. [46], who delayed postharvest fruit and vegetable quality deterioration by optimizing the packaging microenvironment.

Sound fruit percentage is the core intuitive indicator of postharvest storage and transportation preservation efficacy for fruits and vegetables. It directly reflects the degree of mechanical damage and rot deterioration during storage and transportation, serving as an important comprehensive parameter for evaluating packaging preservation performance [47]. Correlation analysis results further revealed the underlying mechanism of postharvest red apricot quality deterioration: during simulated transportation and the entire storage period, sound fruit percentage was significantly negatively correlated with weight loss rate, respiration rate, and MDA content, and significantly positively correlated with fruit firmness, water content, and POD and CAT activities. These findings confirm that excessive water loss, disordered and excessive respiratory metabolism, and aggravated cell membrane oxidative damage are the core drivers of red apricot quality deterioration and reduced marketability during storage and transportation. P1 packaging significantly delays the decline in sound fruit percentage and extends the storage and transportation shelf life of red apricots by effectively regulating the aforementioned key physiological and quality indicators. In addition, total phenols, key non-enzymatic antioxidants in fruits, play a critical role in the antioxidant defense system. Correlation results revealed that fruit total phenol content was significantly positively correlated with POD and CAT activities, and significantly negatively correlated with ROS accumulation. This indicates that total phenols exert a synergistic effect with endogenous antioxidant enzymes to construct a complete fruit antioxidant defense system, effectively alleviate oxidative stress-induced fruit cell damage, and delay postharvest senescence and deterioration. This result is consistent with Wang et al. [48], who confirmed that appropriate preservation treatments increase total phenol content and antioxidant enzyme activities in fruits and vegetables, enhance their endogenous antioxidant capacity, and thereby significantly improve postharvest preservation effects.

The P2 and P3 groups exhibited significantly inferior preservation effects compared with the P1 group, primarily due to inherent limitations in their structural characteristics and material properties. The P2 ventilated foam box has poor heat dissipation [49], which readily causes heat accumulation during storage and transportation. This in turn accelerates fruit respiratory metabolism, exacerbates water loss, and hastens quality deterioration. The P3 small-hole corrugated carton suffers from concurrent deficiencies in heat dissipation and air permeability [50,51]. Poor ventilation and heat dissipation readily induce fruit anaerobic respiration, leading to excessive accumulation of harmful substances such as ethanol, which further aggravates physiological damage and spoilage. In contrast, the P1 plastic crate has excellent air permeability and heat dissipation [52], which effectively improves the storage and transportation environment of red apricots and maintains a stable physiological state throughout the entire cycle. Regarding cushioning performance, all three packaging types rely on primary substrates and matching individual fruit net sleeves for basic protection [53]. The P1 and P3 packages additionally incorporate foam pad cushioning [30], further enhancing mechanical damage resistance. The three packages showed comparable cushioning performance overall. However, Package 1 benefited from its smaller fruit loading capacity. The reduced fruit quantity lowered gravitational impact and further improved its cushioning effect. Taken together, appropriate air permeability, excellent heat dissipation, and sufficient cushioning capacity are the core criteria for selecting postharvest storage and transportation packaging for red apricots. The P1 plastic crate combines all three core advantages. It effectively regulates postharvest physiological metabolism of red apricots, maintains fruit cell structural integrity, and delays storage quality deterioration, as visually evidenced by cell wall ultrastructure. Therefore, it is the optimal packaging method for red apricots under simulated storage and transportation scenarios.

There are two limitations in this study. First, this experiment only compared three commercial packaging methods for red apricots. Given that the main objective was to select the optimal packaging solution for storage and transportation, no unpacked blank control group was included in the experimental design. This study can only reveal the differences among the three packaging treatments, and fails to quantify the comprehensive effects of each packaging type on fresh-keeping and damage resistance when compared with unpacked fruits. Second, all red apricot samples were collected from a single grower with unified field cultivation and management practices. Although samples from a single source ensured uniform maturity, fruit size and initial quality, and guaranteed the reliability of comparative results between groups, they also restricted the generalizability of the research conclusions. Variations in cultivation environment, soil conditions, field management and climate across different producing areas may lead to differences in fruit texture, firmness and mechanical damage resistance. More importantly, distinctions also exist among different apricot cultivars. Further research could be carried out on different apricot cultivars with additional blank control groups in the future.

## 5. Conclusions

In summary, P1 (plastic basket) effectively modulated the physiological metabolism of fresh apricots during simulated transportation and storage. This treatment suppressed accelerated respiration and transpiration water loss, retarded fruit softening, mitigated enzymatic browning and nutrient loss, and sustained the structural stability of pulp cells. By limiting excessive respiration and cell membrane injury, P1 strengthened the antioxidant system of apricot fruit, which increased the proportion of intact fruits and prolonged shelf life. It also slowed down the degradation of soluble solids, titratable acidity and ascorbic acid, relieved lipid peroxidation and oxidative damage. The activities of antioxidant enzymes such as POD and the synergistic action of non-enzymatic antioxidants were well maintained, which reduced the accumulation of reactive oxygen species and delayed senescence. Correlation analysis confirmed that water loss, disturbed respiratory metabolism and oxidative stress were the main causes of quality deterioration in apricots. Equipped with favorable air permeability, heat dissipation and cushioning properties, P1 mitigated the above adverse changes and maintained fruit nutrition and tissue structure. This study elaborated on the influences of different packaging strategies on postharvest apricot quality and relevant physiological mechanisms, offering a theoretical basis and technical references for packaging screening and the improvement of preservation technologies during apricot storage and transportation.

## Data Availability

The original contributions presented in this study are included in the article/Supplementary Materials. Further inquiries can be directed to the corresponding author.

## Supplementary Materials

The following supporting information can be downloaded at https://www.mdpi.com/article/10.3390/foods15122068/s1: The Supplementary Materials include schematic diagrams of the structural design of the three packaging materials (P1, P2, P3) and their stacking states during simulated transportation. Figure S1. Packaging Structure: P1 (plastic basket), P2 (breathable foam box), and P3 (perforated corrugated carton), Figure S2. Packaging stacking method.
