# Supplementary material for: Effects of Different Packaging Methods on the Quality of Fresh Red Apricots During Simulated Transportation and Storage After Transportation
Source: Foods. 2026 Jun 8;15(12):2068. doi: 10.3390/foods15122068 (PMC13297994; doi:10.3390/foods15122068)
Supplement: Supplementary file 1 [file foods-15-02068-s001.zip › foods-4317476-supplementary.pdf]

Supplementary Materials

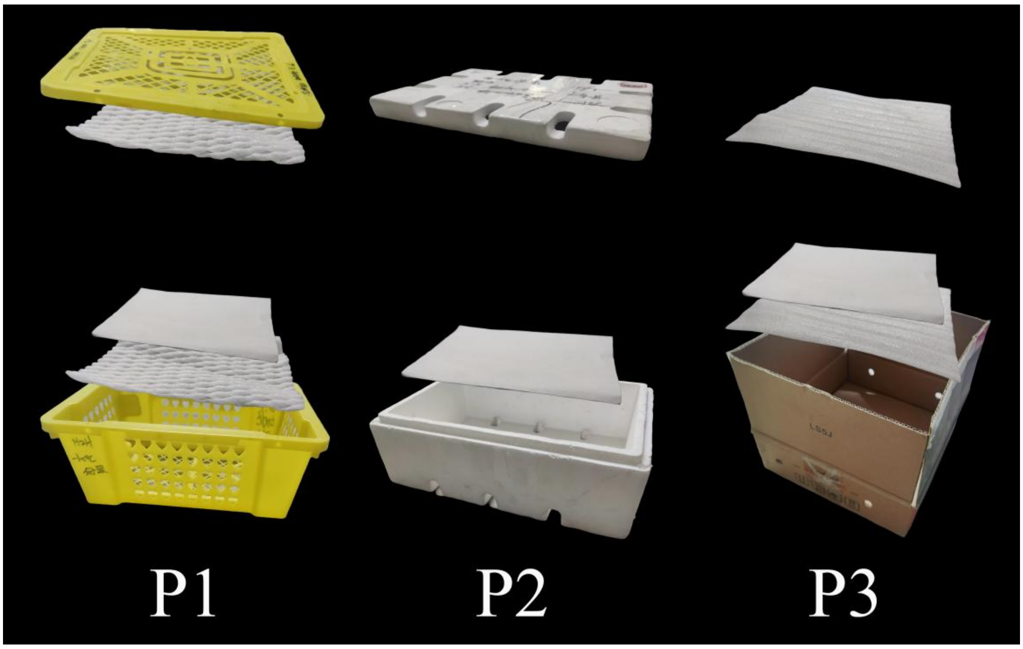

Figure S1. Packaging Structure： P1 (plastic basket), P2 (breathable foam box), and P3 (perforated corrugated carton)

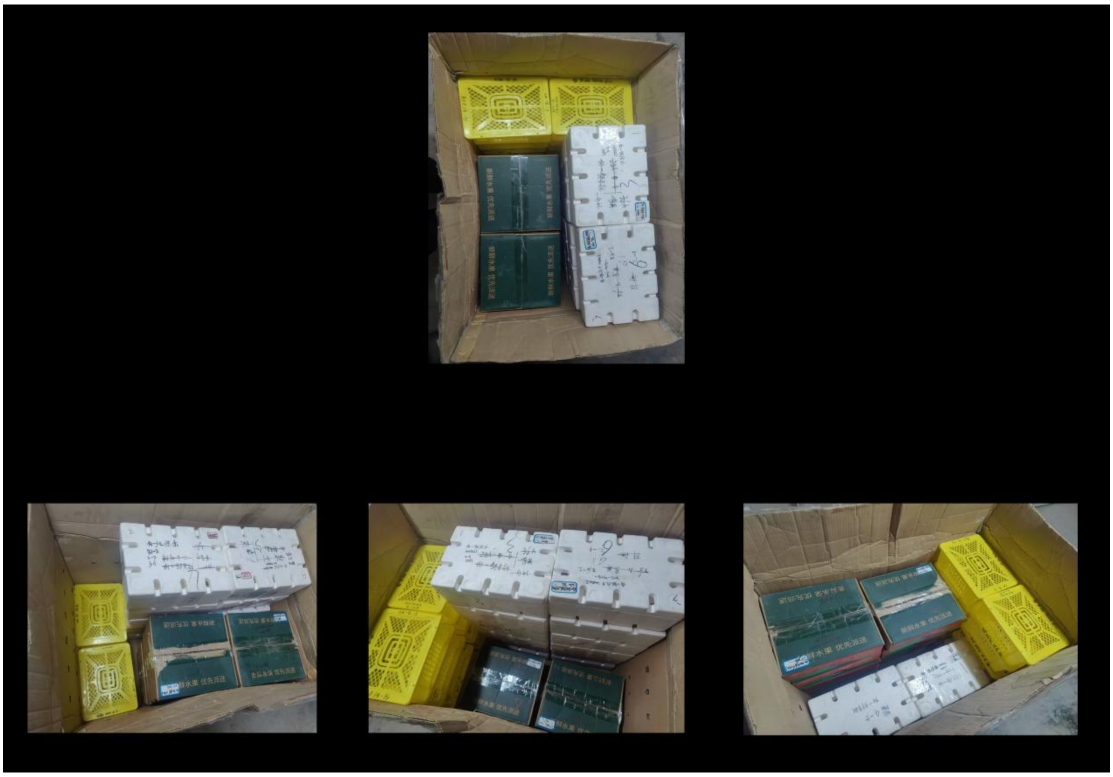

Figure S2. Packaging stacking method
